# Opportunities for antiviral stewardship: a single-center evaluation of maribavir use in solid organ transplant recipients

**DOI:** 10.1017/ash.2026.10865

**Published:** 2026-10-05

**Authors:** Ethan P. Rausch, Alia K. Boland, Kiran Gajurel, Megan D. Shah

**Affiliations:** 1 Division of Pharmacy, Antimicrobial Support Network, Atrium Health Carolinas Medical Center, Charlotte, USA; 2 Transplant Services, Atrium Health Carolinas Medical Center, Charlotte, USA; 3 Infectious Diseases, Atrium Health Carolinas Medical Center, Charlotte, USA

## Abstract

**Objective:**

Maribavir is a novel antiviral for resistant or refractory (R/R) CMV infection with a favorable safety profile. This report describes institutional use of maribavir in solid organ transplant (SOT) recipients with CMV infection to better understand antiviral stewardship opportunities.

**Design:**

Retrospective case series.

**Setting:**

Tertiary referral center.

**Patients/Methods:**

This was a single center case series of adult SOT recipients who received maribavir for treatment of R/R CMV infection. The primary outcome was treatment success by 8 weeks. Treatment failure, including development of maribavir resistance, and CMV recurrence were also assessed.

**Results:**

Maribavir was used for treatment of 23 episodes of CMV infection in 20 SOT recipients. Treatment success was achieved in 14 (60.9%) episodes in unique patients, with 5/14 patients (35.7%) experiencing CMV recurrence within four weeks of maribavir discontinuation. Treatment failure occurred in 9 (39.1%) of 23 treatment courses, with 6/20 (30%) patients developing treatment-emergent maribavir resistance.

**Conclusion:**

Maribavir was a viable option for the management of R/R CMV infection in our real-world single center cohort, as success mirrored that seen in other studies. However, there was a clinically significant portion of patients who failed maribavir or had recurrent CMV. Continued multi-center analysis of maribavir timing, secondary prophylaxis, and patient characteristics that may predict success are warranted to help improve the stewardship of this valuable antiviral.

## Introduction

Antimicrobial stewardship (AS) of solid organ transplant recipients (SOTR) is evolving, including cytomegalovirus (CMV) stewardship, which has the ability to reduce antiviral exposure and CMV resistance.^1^ Management of resistant and refractory (R/R) CMV is particularly challenging with significant antiviral toxicity, morbidity and mortality.^2^ Maribavir, a novel oral benzimidazole with expansive CMV activity without myelosuppression and nephrotoxicity, is an attractive option for management of R/R CMV.^3^ Herein we describe our pragmatic use of maribavir in SOTR to identify practical stewardship opportunities.

## Methods

This retrospective case series included adult SOTR who received maribavir for CMV infection or disease between April 2022 and May 2024 at Atrium Health Carolinas Medical Center. CMV management was non-protocolized, and maribavir prescribing was directed by Infectious Diseases clinicians in collaboration with an AS or SOT pharmacist. Immunosuppression modifications were managed by SOT providers. CMV antiviral resistance testing with genotype sequencing was sent to a reference lab with an expected turnaround time of 4 business days.

The primary outcome was treatment success by 8 weeks (CMV viral load (VL)<200 IU/mL on 2 consecutive occurrences≥5 days apart with symptom resolution). Secondary outcomes included treatment failure by 8 weeks (inability to achieve treatment success, requiring alternative CMV treatment, or development of maribavir resistance) and CMV recurrence (clinically significant DNAemia requiring treatment or CMV disease within 8 weeks after maribavir completion). This study was approved by the local Institutional Review Board.

## Results

We included 23 unique maribavir treatment episodes in 20 SOTR (9 heart, 8 kidney, 2 heart/kidney, and 1 liver). Sixty percent had high-risk CMV serostatus and 10% received treatment for rejection within 6 months prior to CMV case.

Maribavir was utilized for CMV DNAemia in 19 cases (82.6%) and for CMV gastrointestinal disease in 4 cases (17.4%). Maribavir was predominantly used as secondary therapy (87%), most often preceded by valganciclovir (60.9%) and foscarnet (21.7%). Baseline CMV resistance was documented in 11 episodes (47.8%), all of which were UL97 mutations. Patients were switched to maribavir after a median of 27 days, commonly due to neutropenia or leukopenia (47.8%), CMV resistance (26.1%), or fluctuating renal function (13%). Median duration of maribavir was 53 days (IQR 43–60 days).

Clinical success was observed in 14/23 (60.9%) episodes, all in unique patients. Median CMV VL at maribavir initiation was 1,248 IU/mL [IQR 287–5,475 IU/mL] and median time to success was 26 days [IQR 16.5–40.5 days]. Five (35.7%) experienced CMV recurrence within 4 weeks of stopping therapy, of which 3 (60%) developed maribavir resistance with UL97 mutations at T409M, H411Y, and C480F.

Nine episodes (39.1%) in 7 patients were non-responsive to maribavir—3 failed to meet clinical success criteria despite lack of maribavir resistance and 6 developed maribavir resistance during therapy (Table 1). Median CMV VL at maribavir initiation for those who developed resistance on therapy was 7,890 IU/mL [IQR 2,995–19,100 IU/mL].

**Table 1.** Case description of patients experiencing maribavir treatment failure Table 1 long description.

Patient	Transplant organ and CMV serostatus	CMV infection classification	Treatment	Baseline resistance prior to maribavir	CMV VL at maribavir start, IU/mL	Reason for treatment failure	Treatment after maribavir failure
1	Kidney, D+/R−	DNAemia	Secondary	Yes—UL97 M460I, UL54 D413E	1,510	Resistance—UL97 T409M, UL54 K513T	Foscarnet
2	Heart, D+/R−	DNAemia	Secondary	Yes—UL97 L595S	63,900	Increasing VL	High dose ganciclovir
		DNAemia	Secondary	Yes—UL97 L595S	2,600	Resistance—UL97 L595S, H411L mixed mutation	Foscarnet
3	Kidney, D+/R−	DNAemia	Secondary	No	1,640	Failure to reach VL response with persistent low level DNAemia	High dose valganciclovir
4	Heart/kidney, D+/R−	DNAemia	Secondary	Yes—UL97 A594G	46,100	Resistance—UL97 A594G, C480F, H411Y	High dose ganciclovir
5	Kidney, D + R−	DNAemia	Secondary	Yes—UL97 (mixed bases at two nucleotide positions of codon 460), UL54 T503I and P522S	4,180	Resistance—UL97 polymorphism unable to be analyzed	Foscarnet
6	Heart, D+/R+	GI disease with DNAemia	Secondary	Yes—UL97 L595S	11,600	Resistance—UL97 T409M	Foscarnet
		GI disease with DNAemia	Secondary	Yes—UL97 L595S; previous UL97 T409M not detected on repeat resistance testing	2,520	Increasing VL	Foscarnet
7	Heart, D+/R−	DNAemia	Secondary	Yes—UL97 A594V	21,600	Resistance—UL97 T409M	High dose ganciclovir

## Discussion

This case series describes our real-world utilization of maribavir for CMV in adult SOTR who were primarily intolerant or resistant to first-line agents, aligning with international consensus guideline recommendations.^2^ Clinical success (60.9%) was consistent with previously published trials and observational studies.^3–6^

Emergence of maribavir resistance remains a significant concern, which was corroborated in our series, as 39% of episodes led to high level maribavir resistance during or after treatment.^3–6^ Optimal CMV VL to initiate maribavir is an important stewardship consideration. While studies have not shown a significant correlation between baseline CMV VL and maribavir response or development of resistance, there appears to be a declining response to maribavir when initiated at a higher CMV VL.^3^ Some experts recommend initiating maribavir at lower CMV VL, such as <50,000 IU/mL or ideally <5,000–10,000 IU/mL.^2,7^ Given no foscarnet resistance was identified in our cohort and 12/14 (86%) of successful maribavir courses in our study were initiated when CMV VL was <10,000 IU/mL, our data support the transition to maribavir after CMV VL has significantly declined. Our stewardship practice has evolved to start maribavir when CMV VL is <10,000 IU/mL, whenever feasible

Despite initial success, 35% of patients developed CMV recurrence within 4 weeks of discontinuing maribavir, aligning with findings from other observational cohorts (33%–45%), but higher than observed in the randomized clinical trial (26%).^3–6^ Management after maribavir treatment success was heterogenous, as some continued maribavir treatment despite response, discontinued therapy, or implemented secondary prophylaxis, highlighting areas for stewardship intervention. In the 3 patients who developed recurrence with maribavir resistance, maribavir was extended for a median of 33 days after achieving initial treatment success. Perhaps continued antiviral exposure, despite low VL, placed additional selective pressure and increased resistance risk. ^8^ While none of our patients had a CMV-cell mediated immunity (CMV-CMI) assay, CMV-CMI monitoring is a promising opportunity for AS to individualize duration of therapy and secondary prophylaxis, as it may reduce relapse and recurrent CMV. ^9^ Lastly, secondary prophylaxis with letermovir may be another intriguing option as it has been associated with corresponding acquisition of CMV-CMI.^10^

This study is limited by its retrospective and single center design, small cohort, single electronic health record, and lack of comparator group precluding inferential analysis. We were unable to assess immunosuppression modifications during CMV treatment, which may influence treatment outcomes or resistance acquisition. Lastly, given the lack of an institutional CMV treatment protocol, CMV management remains heterogenous. Utilization of adjunctive therapies, treatment duration, and decision to use secondary prophylaxis, was at the discretion of the treating provider, which may influence outcomes.

Maribavir is an important therapeutic advancement in the management of R/R CMV infection. Ongoing AS efforts focused on treatment timing, immune guided-management, and duration optimization are paramount to maximizing treatment success and preserving maribavir’s efficacy.

## Table 1. Long description

The table has seven rows and seven columns. The columns are labeled: Patient, Transplant organ and CMV serostatus, CMV infection classification, Treatment, Baseline resistance prior to maribavir, CMV VL at maribavir start, IU/mL, Reason for treatment failure, and Treatment after maribavir failure. Each row provides specific details for seven patients. For example, Patient 1 has a kidney transplant, D+/R- serostatus, DNAemia infection classification, secondary treatment, baseline resistance with UL97 M460I, UL54 D413E, CMV VL at 1,510 IU/mL, resistance reason with UL97 T409M, UL54 K513T, and foscarnet treatment after maribavir failure.

Navigate back to Table 1..
